# What’s next for osteoarthritis gene therapy?

**DOI:** 10.3389/fbioe.2026.1871809

**Published:** 2026-07-06

**Authors:** Christopher H. Evans, Steven C. Ghivizzani, Annahita Keravala, Thomas W. Chalberg, Paul D. Robbins

**Affiliations:** 1 Musculoskeletal Gene Therapy Laboratory, Mayo Clinic, Rochester, MN, United States; 2 Department of Orthopedics and Rehabilitation, University of Florida School of Medicine, Gainesville, FL, United States; 3 Genascence Corp., Palo Alto, CA, United States; 4 Department of Biochemistry, Molecular Biology and Biophysics, University of Minnesota School of Medicine, Minneapolis, MN, United States

**Keywords:** arthritis, biological therapy, clincial trials, intra-articular (IA) delivery, research translation, viral vector

## Abstract

Interest in using gene therapy to treat osteoarthritis (OA) is growing and a number of clinical trials have been initiated. This commentary identifies three intersecting areas that need to be addressed for the field to move forward expeditiously. The first relates to lowering the cost of manufacturing clinical grade viral vectors, addressing various aspects of their deployment, and overcoming immune barriers to dosing and re-dosing. The second area requires an improved understanding of the pathophysiology of OA, including its stratification by endotype and phenotype. Coupled to the development of reliable biomarkers, this will enable the creation of personalized gene therapies, facilitate patient selection, and aid the identification of additional molecular targets. Moreover, progress in the early diagnosis of OA will enable administration of gene therapeutics at a stage when they are most likely to be successful. Finally, important issues with regard to financing and regulation are discussed. Top line data from two pivotal Phase III clinical trials are expected to be released this year. The findings from these trials will exercise considerable influence on the future development of the field.

## Introduction

Osteoarthritis (OA) is incurable, difficult to treat and highly prevalent, affecting nearly 600 million individuals world-wide (GBD 2021 Osteoarthritis Collaborators, 2023). The introduction of anti-arthritic genes into joints with OA is a novel approach to treating this recalcitrant disease. After a long lag phase and a couple of false starts, the field of OA gene therapy is finally gaining traction (Evans et al., 2026) and there is increasing interest in this previously overlooked area (Evans et al., 2025). With an increasing number of clinical trials (Table 1) and the pending release of pivotal Phase III data, the field is approaching an inflection point. This provides a timely opportunity to review the present state of affairs and identify key points to consider for future success.

**TABLE 1:** Clinical trials in the gene therapy of osteoarthritis.

Transgene	Product name	Vector (*In/Ex vivo*)	Phase	NCT identifier	Status per Clinicaltrials.gov	Refs
TGF-β_1_	TG-C (Formerly Invossa)	Retrovirus *Ex vivo*	I I II IIa IIb III III III II I	02341391 00599248 01221441 02341378 01671072 02072070 03291470 03203330 05276011 * 06144970**	Completed Completed Completed Completed Completed Completed Active, not recruiting Active, not recruiting Not yet recruiting Not yet recruiting	Kim et al. (2018), Lee et al. (2020), Ha et al. (2012)
IFN-β	ART-102	AAV *In vivo*	I	02727764***	Unknown	Vrouwe et al. (2022)
IL-1Ra	GNSC-001	AAV *In vivo*	I Ib	02790723 05835895	Completed Active, not recruiting	De la Vega et al. (2025)
IL-1Ra	PCRX-201 (formerly FX201)	HC-Adenovirus *In vivo*	I II	04119687 06884865	Active, not recruiting Active, not recruiting	Kelley et al. (2021)
IL-10	XT-150	Plasmid *In vivo*	I I I II	03477487 3769662 03282149 04124042	Completed Completed Completed Completed	​
NKX3.2	ICM-203	AAV *In vivo*	I/IIa I/II	04875754 05454566 05752032	Active, not recruiting Not yet recruiting Enrolling by invitation	Heald et al. (2024)
“optimized human anti-inflammatory protein”#	BBM-A101	AAV *In vivo*	I	07198555	Recruiting	​

Modified from reference 3. All trials target the knee joint, apart from:

* hip.

** intervertebral disc.

*** finger joints.

# according to the company website: https://www.beliefbiomed.com/en/newsd-824.html

## Present state of the field

A number of sponsors are actively pursuing gene and genetically-modified cell therapies for OA with several clinical trials completed and others in progress (Table 1) (Evans et al., 2023). The favored strategy involves delivering cDNAs encoding therapeutic gene products to individual joints by intra-articular injection (Bandara et al., 1992). This can be achieved by either *in vivo* or *ex vivo* technologies*,* and a variety of viral and non-viral vectors have been explored for this purpose in pre-clinical models. Present clinical trials (Table 1) use adeno-associated virus (AAV), high capacity adenovirus (HC-Ad), or plasmid DNA for *in vivo* delivery and γ-retrovirus for *ex vivo* delivery via transduced cells.

No genetic therapy for OA has marketing approval, but results from two pivotal, Phase III trials of TG-C are expected to be announced this year and, if successful, could lead to a subsequent biologics license application (BLA) to the US FDA. TG-C is made using cells transduced *ex vivo*. The first clinical trial for gene transfer to a human joint also used an *ex vivo* protocol in which autologous synovial fibroblasts were retrovirally transduced with cDNA encoding the interleukin-1 receptor antagonist (IL-1Ra) and injected into metacarpophalangeal joints with rheumatoid arthritis (RA) (Evans et al., 2005). Although this Phase I clinical trial was successful, its strategy was abandoned due to the cost and complexity of *ex vivo* gene transfer using culture-expanded autologous cells, and concerns about insertional mutagenesis using retroviral vectors (Evans et al., 2026). Nevertheless, Kolon TissueGene developed a modified version of this technology which, after some restructuring, uses an established human cell line (HEK293), retrovirally transduced to express transforming growth factor (TGF)-β_1_. The transduced cells are irradiated at a dose that inhibits cell division, and hence malignancy, but does not restrict TGF-β_1_ production. These cells are mixed with chondrocytes, originally derived from a young patient with polydactyly, before injection into joints with OA (Kim et al., 2018; Lee et al., 2020; Ha et al., 2012). This protocol has completed two pivotal, phase III trials whose top line data are expected to be announced later in 2026. Several phase II trials using *in vivo* transgene delivery are in progress (Table 1) and a pivotal phase II/III trial of GNSC-001 has been cleared by the FDA.

The pre-clinical pipeline is robust, with various transgenes being tested in animal models, including those encoding a variety of cytokines, cytokine antagonists, growth factors and lubricin, among others, as well as non-coding RNA, reprogramming factors and gene editing components. Most, but not all, of these studies use AAV as the gene delivery vehicle. Additional vectors investigated in pre-clinical studies of *in vivo*, intra-articular gene transfer include γ-retrovirus (Ghivizzani et al., 1997), lentivirus (Gouze et al., 2003; Gouze et al., 2002; Huang et al., 2012), herpes simplex virus (Levitt et al., 2025; Oligino et al., 1999), and various non-viral formulations (Fu et al., 2025; Zhang et al., 2024; Keravala et al., 2006).

## Issues for future development

### Vector manufacture and deployment

One consistent challenge in the gene and cell therapy field is the high cost of manufacturing viral vectors under current Good Manufacturing Practice (cGMP) regulations. When administered systemically, AAV vectors are dosed in the range of 2 × 10^13^–1 × 10^14^ viral genomes (vg)/kg, which could reach 1 × 10^16^ total vg dose in an adult patient. The cost of manufacturing at this dose can exceed $1 million using traditional manufacturing methods including the considerable expense of cGMP adherence. Such approaches are possible for products treating a rare disease that can command high prices, such as Zolgensma for spinal muscular atrophy ($2.1 million) and Hemegenix for hemophilia ($3.5 million), but are not feasible for major diseases like OA. Efforts to improve manufacturing processes as well as the potency and efficiency of existing vectors could make a meaningful contribution toward lowering costs.

Importantly, whereas rare disease gene therapies are delivered systemically, intra-articular gene therapy is, in contrast, delivered locally and thus requires orders of magnitude less vector. Intra-articular AAV therapies in the knee, for example, are dosed in the range of 1 × 10^12^–1 × 10^13^ total vg, which is 1,000–10,000 fold lower than doses used systemically. Because the therapies being evaluated in clinical trials (Table 1) use a variety of vectors and strategies, it is not possible to generalize about pricing, but a reasonable goal would be for costs to be competitive with those of rival treatments and procedures such as total joint arthroplasty.

Non-viral vectors are less expensive than their viral counterparts, but are also far less efficient. When injected into joints they typically provide transient, low levels of gene expression; many formulations are inflammatory. However, newer nanoparticle formulations, including dendrimers (Geiger et al., 2018), certain liponanoparticles (LNPs) (Dewani et al., 2026) and derivatized extra-cellular vesicles (Pathrikar et al., 2025), show promise as agents that penetrate articular cartilage and deliver payloads to chondrocytes *in situ*. The development by Vedadghavami et al. (2019) of cartilage penetrating cationic peptide carriers has greatly aided these endeavors. None of these formulations have been tested in human joints but preclinical data are encouraging. Plasmid DNA encoding a variant of IL-10 has completed a phase II clinical trial (Table 1).

One impediment to the wider use of virally based gene therapies is the need for long-term storage in ultra-low temperature freezers (≤80 °C). Although these are used in certain care centers such as hospital pharmacies, they are not widespread in most clinics providing routine care for OA patients. Alternative approaches, such as long-term storage at ≤ 80 °C centrally and short-term refrigeration or room temperature storage locally, are being developed to increase availability. Recent data suggest that lyophilization with cryopreservants also holds promise as a way to maintain the potency of multiple serotypes of AAV at higher temperatures (Zhang et al., 2021; McGlinch et al., 2025). Lyophilization is also being explored as a way of maintaining the potencies of adenovirus and other vectors at temperatures above −80 °C (Chen et al., 2021).

Viruses are antigenic and, because those being developed as vectors for human gene therapy exist in nature, a proportion of individuals who are candidates for gene therapy have pre-existing antibodies, including neutralizing antibodies (NAbs), to the viral vector. This varies by virus, serotype, and geographic location. Recent measurement of preexisting NAbs in serum and synovial fluid aspirated from human joints with OA in preparation of a clinical trial using AAV2.5 found that in approximately 66% of patients, NAb titers were low or undetectable (Abdul et al., 2023). Only 16% had pre-existing, high titer NAbs to the AAV2.5 capsid. Regardless of pre-existing NAbs, intra-articular injection of AAV into human knees stimulates a robust humoral response, with high-titer NAbs present in serum and synovial fluid (De la Vega et al., 2025). However, no cell-mediated immune response has been detected. It is not known whether the presence of high titer NAbs in synovial fluid impairs dosing or re-dosing in joints. The fact that NAb titers in synovial fluid correlate with those in serum provides the basis for a simple blood test when screening NAb titers in prospective patients (De la Vega et al., 2025). A similar state of affairs obtains for adenovirus vectors, with NAbs to serotypes 5, 6, 26 and 36 widely present in the sera of the majority of populations in different countries (Mast et al., 2010). The degree to which the intra-articular injection of adenovirus vectors induces cell-mediated responses or NAbs in human synovial fluid and serum, has not been reported.

Immune responses to vectors are an issue throughout the whole field of gene therapy, with transient immunosuppression being explored widely as one means of allowing efficient dosing without eliciting a strong immune response. In the case of a non-lethal disease like OA, such regimens must balance benefit-to-risk and carry an acceptable cost. Intra-articular or oral steroids provide one possibility, with the advantage that they are already used for treating OA. An alternative approach shields the virus from the immune system by encapsulating it within an exosome or LNP (Maguire et al., 2012). Preliminary data concerning the use of enveloped AAV in the context of delivery to articular cells are encouraging (Atasoy-Zeybek et al., 2026). In larger joints, such as the knee, it may be possible to lavage the joint to remove NAbs present in synovial fluid for a sufficient length of time to permit efficient transduction.

### Improved understanding of the biology of osteoarthritis

OA is a heterogeneous disease with different phenotypes and endotypes (Mobasheri et al., 2026). Thus it is highly unlikely that a single gene therapeutic will be equally effective in all patients and all subtypes of disease. As the molecular and biological stratification of patients with OA improves, it should be possible to design more precise, endotype- or phenotype-specific gene therapies with high efficacy in defined subsets of patients.

Increasing knowledge of the pathophysiology of OA may also identify additional therapeutic gene products. Present gene therapies (Table 1) deliver a single transgene to the patient. The possibility that vectors co-delivering two or more therapeutic genes have greater potency has not been widely explored. However, enhanced potency in pre-clinical models of OA using combinations of IL-1Ra with lubricin (Stone et al., 2019), soluble tumor necrosis factor receptor 1 (Wang et al., 2006) or Sox9 (Zhou et al., 2025) has been reported. In this context, co-delivery of IL-1Ra with a growth factor gene that regenerates cartilage is an attractive future strategy. However, delivering cDNAs encoding growth factors raises concerns of adverse effects resulting from prolonged or excessive transgene expression. Under these circumstances regulated gene expression may be required so that the growth factor is produced episodically at controlled levels.

It is also possible to consider combination therapy in which a gene therapeutic is delivered with a more traditional pharmaceutical. Steroid is an obvious choice for intra-articular therapy and is being used in the Phase II trial of PCRX-201 to diminish the inflammatory response to the adenovirus. Steroid has also been co-administered with AAV in certain patient cohorts in the Phase Ib trial of GNSC-001.

Another matter for future development is the treatment of joints other than the knee. Although the knee is an obvious first target, there has been little attention paid to other joints where OA is prevalent. ART-102 was trialed in the small joints of the hand, and TG-C has trials pending in the hip and intervertebral disc. The degree to which the pathophysiology of OA differs in important details between different joints is unknown, so outcomes may not necessarily be the same. For instance, it is estimated that up to 50% of hip OA results from femoro-acetabular impingement (Heerey et al., 2026).

The field would be greatly aided by the identification of reliable biomarkers that could be used for diagnosis, monitoring of disease progression and, once specific endotypes have been confirmed, guiding patient selection. If OA could be diagnosed early, even before symptoms have become obvious, the gene therapeutic could be administered at a stage where it has the greatest chance of success.

### Funding and regulatory landscape

Although the concept of an intra-articular gene therapy for OA is now well established, its continued development requires continued funding for both additional pre-clinical research and clinical validation. These needs coincide with a time when, in the USA, federal funding for research is under threat and commercial investment in gene therapy as a whole is tepid (Albert, 2026).

Clinical development is expensive and therefore in the hands of commercial entities, often small start-up biotechnology companies that rely on investors for continuing their activities. Before committing funds, investors require good evidence of efficacy and, especially, safety for a gene therapy product treating a common, non-lethal disease such as OA. Thus the field suffered an early set back when a patient died in a Phase II clinical trial that used intra-articular AAV to treat RA (Evans et al., 2008; Frank et al., 2009). A subsequent investigation by the FDA exonerated the gene therapeutic and since then there have been no further incidents of this type. However, a Phase I clinical trial in which AAV was used to deliver interferon-β to finger joints with inflammatory arthritis, including OA, was stopped recently because of persistent, severe tenosynovitis (Vrouwe et al., 2022).

Under these circumstances perhaps the single, biggest, immediate boost to the further development of the OA gene therapy field would be robust demonstration of efficacy and safety in a well-powered clinical trial. Thus results from the two Phase III studies expected to be announced in the second half of 2026 will have a powerful influence on the future of the field. Although the product in question, TG-C, is idiosyncratic, failure to meet its primary endpoint or a troubling safety signal could set back the field. Conversely, confirmation of efficacy and safety, followed by a successful BLA could provide an important boost.

Finally, in February 2026 the FDA announced their new default position of requiring only a single pivotal trial for drug approvals instead of the previous default position of requiring two such trials (Prasad and Makary, 2026). This will simplify the path to clinical approval and lower development costs for all drugs, including genetic medicines for OA.

## Discussion

With pre-clinical proof of concept established and advanced clinical trials underway, OA gene therapy is at an important juncture. To attract the resources needed for its further development, the clinical trials that are presently in process need to demonstrate safety and meet their primary clinical end points in a robust manner. This is particularly true of the two Phase III trials of TG-C whose top line data should be announced this year. Although this product is atypical and *ex vivo*, its success will encourage development of newer genetic medicines and encourage the investment that is necessary before outstanding issues can be resolved. These include addressing neutralizing immune reactions and lowering manufacturing costs, while improving the performance of existing vectors, developing novel vectors, identifying additional therapeutic transgenes and exploring combination therapies. These activities should take place in concert with an evolving understanding of the biology and pathophysiology of OA in order to foster the development of individualized gene therapies as specific endotypes and phenotypes are identified. The verification of robust biomarkers will aid greatly these endeavors, especially if they permit OA to be identified at an early stage, even before symptoms become apparent. Under these conditions, gene therapies could be administered at a time when they have the greatest opportunity to succeed. Given the high global prevalence of OA, its enormous economic and social cost, and its therapeutic recalcitrance, these are attractive possibilities.
